# Potential Toxicity and Underlying Mechanisms Associated with Pulmonary Exposure to Iron Oxide Nanoparticles: Conflicting Literature and Unclear Risk

**DOI:** 10.3390/nano7100307

**Published:** 2017-10-06

**Authors:** Tiffany G. Kornberg, Todd A. Stueckle, James M. Antonini, Yon Rojanasakul, Vincent Castranova, Yong Yang, Liying W. Rojanasakul

**Affiliations:** 1 Pharmaceutical and Pharmacological Sciences, West Virginia University, Morgantown, WV 26505, USA; yrojan@hsc.wvu.edu (Y.R.); vcastran@hsc.wvu.edu (V.C.); 2 Health Effects Laboratory Division National Institute for Occupational Safety and Health, Morgantown, WV 26505, USA; jux5@cdc.gov (T.A.S.); jga6@cdc.gov (J.M.A.); 3 Department of Biomedical Engineering, University of North Texas, Denton, TX 76207, USA; yong.yang@unt.edu

**Keywords:** iron oxide, nanoparticles, toxicity, *in vivo*, *in vitro* model

## Abstract

Fine/micron-sized iron oxide particulates are incidentally released from a number of industrial processes, including iron ore mining, steel processing, welding, and pyrite production. Some research suggests that occupational exposure to these particulates is linked to an increased risk of adverse respiratory outcomes, whereas other studies suggest that iron oxide is biologically benign. Iron oxide nanoparticles (IONPs), which are less than 100 nm in diameter, have recently surged in use as components of novel drug delivery systems, unique imaging protocols, as environmental catalysts, and for incorporation into thermoplastics. However, the adverse outcomes associated with occupational exposure to IONPs remain relatively unknown. Relevant *in vivo* studies suggest that pulmonary exposure to IONPs may induce inflammation, pulmonary fibrosis, genotoxicity, and extra-pulmonary effects. This correlates well with *in vitro* studies that utilize relevant dose, cell type(s), and meaningful end points. A majority of these adverse outcomes are attributed to increased oxidative stress, most likely caused by particle internalization, dissolution, release of free iron ions, and disruption of iron homeostasis. However, because the overall toxicity profile of IONPs is not well understood, it is difficult to set safe exposure limit recommendations that would be adequate for the protection of at-risk workers. This review article will focus on known risks following IONPs exposure supported by human, animal, and cell culture-based studies, the potential challenges intrinsic to IONPs toxicity assessment, and how these may contribute to the poorly characterized IONPs toxicity profile.

## 1. Introduction

The field of nanotechnology is expanding rapidly as scientists and engineers continue to develop innovative applications using nano-scaled materials. For example, iron oxide nanoparticles (IONPs) have been developed for use in targeted cancer treatment, new imaging techniques, and as environmental catalysts due to their unique paramagnetic properties [1]. They can be incorporated into thermoplastics and other materials due to their pigmented properties, as well [2]. Iron oxide particulates (both fine/micron- and ultra-fine/nano-sized) are known to become aerosolized during anthropogenic activities related to the iron and steel industries, as well as during the nanoparticle manufacturing process, where they may represent a significant portion of the circulating air for industrial workers [3,4]. However, the actual adverse outcomes to human health induced by iron oxide particulate exposure remain highly controversial. The most common adverse health outcomes induced by iron oxide include permanent discoloration of the eyes, siderosis, and pneumoconiosis [5]. Some epidemiology studies also indicate adverse respiratory outcomes and increased risk of lung cancer following inhalation of iron oxide particulates in an occupational setting [3,5,6,7], whereas other researchers report iron oxide particulates to be biologically benign [8,9,10,11,12,13]. The exact mechanisms underlying iron oxide particulate-induced adverse outcomes are poorly understood, but most research suggests they are related to the physicochemical properties of the iron or iron oxide metal compounds, and are largely due to iron oxide-induced or catalyzed oxidative stress. 

Iron is a transition metal, meaning it exists in several different valence states, and can form compounds with other elements such as oxygen. Iron oxides come in three main oxidative states: FeO, Fe_2_O_3_, and Fe_3_O_4_. FeO (wustite) makes up a significant component of the earth’s mantle and may have electrical conducting properties [14]. It is also used as a pigment for tattoo inks [15], and is involved in the forge welding process. Fe_2_O_3_ (hematite) is ferromagnetic, and has two common structural forms. α-Fe_2_O_3_ is rhombohedral and is the most common type of iron oxide mined during the iron ore mining process [16]. At high temperatures, α-Fe_2_O_3_ is converted to the gamma form (maghemite), which has a cubic structure. γ-Fe_2_O_3_ becomes superparamagnetic at sizes of 10 nm or less, making it relatively popular for biomedical applications. Fe_3_O_4_ (magnetite) is ferrimagnetic and was a primary component in rudimentary forms of the compass [17]. On the nano-scale, Fe_3_O_4_ will take on paramagnetic or superparamagnetic properties, which also allow it to be utilized in a broad range of applications.

**Figure 1:**
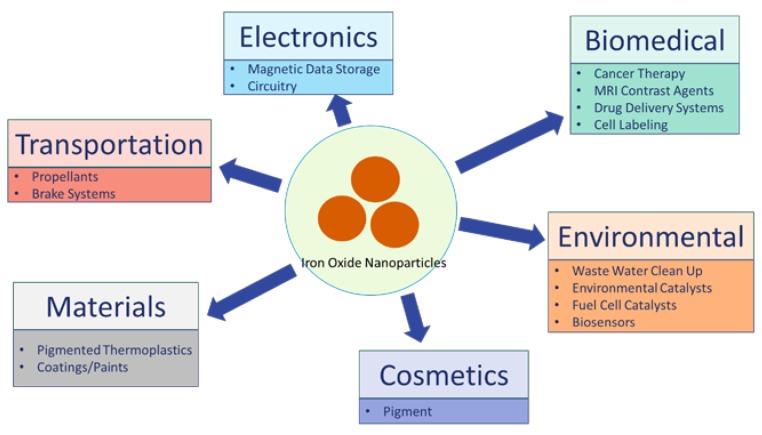
IONPs Uses by Humans and Potential Exposure Sources. IONPs are currently being used in a wide range of fields, including biomedical, electronic, transportation, environmental, materials, cosmetics, and more. Currently, biomedical uses for IONPs, including cancer therapy, MRI contrast agents, and targeted drug delivery systems may involve injection into humans. Cosmetics products may involve dermal application. IONPs use in propellants, or as pigmented components of coatings/paints may result in pulmonary exposure. However, adverse outcomes resulting from these exposures remain largely unknown.

Examples of applications that rely on micron/fine-sized iron oxide include: medicine, electronic tape, pigments, and catalysts. Both γ-Fe_2_O_3_ and Fe_3_O_4_ can be purposefully engineered to be less than 100 nm in diameter, and possess unique paramagnetic or superparamagnetic properties at sizes of 10 nm or less. Because of this, IONPs are being incorporated into a wide range of consumer products and industrial applications [18], including biomedical (cell labeling, targeted drug delivery platform, and novel imaging techniques), electronics (circuitry, data storage), transportation (brake systems), and cosmetics (Figure 1). 

Although IONPs are involved in a broad range of applications, their toxicological profile remains unclear. This review will first focus on what is known of IONPs toxicity based on representative human, *in vivo*, and *in vitro*-based studies. Representative epidemiology studies will be used to highlight potential adverse outcomes induced by IONPs exposure based on fine/micron-sized iron oxide reports (summarized in Table 1), followed by *in vivo* and *in vitro* studies to better ascertain IONP-induced adverse outcomes, as well as the potential underlying mechanism. Then, intrinsic issues with IONPs toxicity assessment will be addressed, and further clarified how these issues may contribute to the lack of certainty regarding IONP-induced adverse outcomes. This review is not meant to be a comprehensive review of IONPs toxicity literature, but rather a critical evaluation of the literature that does exist, and why so much of it appears to be conflicting.

## 2. Potential Toxicity of IONPs Based on Human Studies

### 2.1. Mining/Industrial Applications—Fine/Unknown-Sized Iron Oxide

Human exposure to iron oxide particulates, especially for miners and industrial workers, has been relatively well studied. There are a wide range of industries that involve worker exposure to fine/micron-sized iron or iron oxide particulates. These include iron ore mining and pyrite production, as well as industries which use iron oxide as a pigment or polishing agent, or which involve the mechanical alteration of iron containing products [19]. These processes release iron oxide particulates into the circulating air, becoming an exposure risk for workers, including miners, foundry workers, smiths, and metal grinders [7,12]. An occupational exposure is more likely to be continuous or extended [7], thus potentially leading to exacerbated risk of chronic health conditions. However, iron or iron oxide dusts often contain quartz or silica [10], as well as mixtures of other workplace particulates (e.g., welding), which make it difficult to distinguish iron oxide-specific effects. Furthermore, the size of the iron oxide released during these processes often varies greatly. Therefore, the actual adverse outcomes induced by iron oxide particulates in these settings remain controversial and unclear.

Iron ore mining is known to release iron oxide dust, and many researchers have focused on this occupation to elucidate the potential adverse outcomes associated with iron oxide particulate exposure. Boyd et al. [6] found that underground iron ore miners had an almost 75% increased risk of lung cancer related death as compared to local, non-mining subjects. The authors attributed these deaths to the potentially carcinogenic effect of iron oxide. Studies which followed iron ore miners in both Finland [20] and China [21] reported similar outcomes. A lack of ventilation during the mining process, which leads to increased particulate exposure [21], was used as additional evidence that the iron oxide particulates released during the mining process were most likely responsible for subsequent lung cancer-related deaths. However, these three studies also note that an excessive amount of radon daughters, as well as the inability to fully characterize particulate size and composition in the circulating air, made it difficult to conclusively attribute the observed increased risk of lung cancer to iron oxide exposure, specifically.

Currently, the Occupational Safety and Health Administration (OSHA) has a permissible exposure limit of 14 mg/m^3^ for fine iron oxide over the course of an 8 h work day. The National Institute for Occupational Safety and Health (NIOSH) set a recommended exposure limit (REL) of 5 mg/m^3^ for iron (in iron oxide) over a 10 h work day, while the American Conference of Governmental Industrial Hygienists (ACGIH) recommended 5 mg/m^3^ limit for the respirable fraction of iron oxide over an 8 h workday [5]. However, there is currently no separate REL for IONPs due to lack of toxicity and hazard assessment, which may put vulnerable workers at risk of IONP-induced adverse outcomes. This has become a more critical problem recently, due to the rapid development and projected influx of IONP-based applications.

### 2.2. Ultrafine and Nano-Sized Iron Oxide

IONPs are currently being incorporated into a wide range of consumer products. As applications for IONPs expand, there is an increasing concern for potential toxicity induced by these materials, especially for those who would be exposed in an occupational setting. It is generally believed that decreasing particle size leads to increasing toxicity on a per mass basis due to increased particle surface area and number [22,23]. Therefore, IONPs may have increased potential to induce adverse health effects as compared to their fine/micron-sized counterparts. However, there is currently very little known about these potential risks. The limited human data on IONPs toxicity largely focuses on either occupational exposures to particulate mixtures, which are known to include iron or iron oxide (such as welding, iron smelting, or steel processing), or workers in IONPs manufacturing facilities. Representative studies are presented below, and are summarized in Table 2.

There are a large number of mixed particulate exposures that contain iron or iron oxide particulates, including certain types of welding fumes. Andujar et al. [24] used a cohort of 21 welders to assess welding fume exposure as a potential cause for observed lung changes in patients. The authors collected lung samples from 21 welders who had worked for an average of 27 years each, and compared the tissue to similarly obtained lung samples from 21 matched controls. The welders were shown to have increased fibrotic lesions and elevated lung iron levels as compared to non-welders. This was shown in conjunction with iron oxide, manganese oxide, and chromium oxide particulates internalized within alveolar macrophages. When the authors synthesized representative nanoparticles (including Fe_2_O_3_ and Fe_3_O_4_) and used them to treat human macrophages *in vitro*, the IONPs were found to induce pro-inflammatory cytokine secretion [24]. This directly implicates iron oxide as being a driver for an inflammatory response, which when dysregulated or dysfunctional, can be known to contribute to pulmonary fibrosis as was observed in these welders. 

IONPs are also specifically engineered for a wide range of consumer products and applications, leading to the recent increase of IONPs manufacturing facilities. Unfortunately, due to the relatively high exposure limit recommendation for fine iron oxide, along with the greater toxic potential of nano-sized particulates in general, these types of facilities have the potential to provide much better evidence for IONP-specific-induced adverse effects. Xing et al. [3] assessed the concentration of airborne nanoparticles in a factory which manufactured Fe_2_O_3_-based materials via chemical synthesis. Air sampling locations were chosen based on proximity to one of four major steps involved in this synthesis process, and included two packaging locations, a powder screening location, and a material feeding area. The authors found a significantly higher amount of Fe_2_O_3_ between 10 and 1000 nm in diameter in these four locations as compared to both indoor and outdoor background particulate levels. They reported an average (across all four sites) of three fold increased number of particles, two fold increase in mg/m^3^ particle concentration, and almost two fold increase in total particle surface area as compared to background particulate levels. Total exposure ranged from 0.04 to 0.28 mg/m^3^ depending on site, which is much lower than the previously described OSHA permissible exposure limit (14 mg/m^3^ for fine iron oxide over an 8-h work day). However, the authors did not assess if any adverse health outcomes were reported in these exposed workers.

Pelclova et al. [4] assessed workers known to be exposed to an iron oxide aerosol (80% of which was less than 100 nm in diameter) in a nanoparticle manufacturing facility. Median mass exposure concentrations were 0.083 mg/m^3^, or 66,800 particles/cm^3^ (lower than the OSHA permissible exposure limit, but roughly similar to the Xing et al. [3] reported exposure) with an average of 10 years exposure per worker. Workers were evaluated at the end of a work shift, and were found to have elevated oxidative stress and inflammatory biomarkers in exhaled breath condensate and urine, indicating the potential for IONPs to induce adverse outcomes with a long-term occupational exposure. A caveat to this study, however, is the transient nature of oxidative stress biomarkers in the urine. A more thorough evaluation of these workers, as well as a larger sample size, would be necessary to definitively illustrate the potential systemic oxidative stress response induced by IONPs occupational exposure, and to better link this response to subsequent adverse health outcomes.

Overall, these studies provide direct evidence that workers involved in the IONPs synthesis or manufacturing process are exposed to IONPs in an occupational setting, and that this exposure can lead to oxidative stress and inflammation over an extended period of time. Due to the limited amount of information available, however, no exposure limit recommendations for IONPs have been made, and both its full toxicity profile and the underlying mechanisms of action are still poorly understood.

## 3. Toxicity of IONPs—*In Vivo* Studies

*In vivo* toxicity testing is currently the primary source of information to establish potential risk following particle exposure. The observed animal response can be extrapolated to risk of adverse outcomes in humans, and is used to help establish or support safe exposure limit recommendations. The most common route of IONPs exposure in an occupational setting is via inhalation. However, very few *in vivo* studies have been done which assess this route for IONP-induced toxic effects. Furthermore, the resulting data often show conflicting results, or are otherwise inconclusive. The studies which do report adverse outcomes following IONPs pulmonary exposure reveal subsequent inflammation [25,26,27,28,29,30], pulmonary fibrosis [31,32], genotoxicity [33,34], and extra-pulmonary effects [35,36,37], almost all of which are attributed to IONP-induced excessive oxidative stress. Representative studies are presented below, and are summarized in Table 3.

### 3.1. Inflammation

The inhalation of a foreign particle will likely induce an inflammatory response immediately following exposure. This allows lung immune cells to identify and remove foreign material from the lung, in an attempt to minimize tissue injury and damage. If the particle persists and inflammation becomes prolonged, it may become dysfunctional and can ultimately lead to development of fibrosis, cancer, or other adverse outcomes [38,39]. IONPs have been shown to induce an acute inflammatory response in both rats and mice, which in some cases may become prolonged or chronic [26]. IONPs may also modulate a pulmonary allergic reaction [29,30].

Park et al. [25] exposed ICR mice via a single intratracheal instillation to Fe_3_O_4_ (5.3 nm primary particle size) at the dose of 250, 500 μg/kg, or 1 mg/kg (about 0.005 to 0.02 mg/mouse), and assessed the inflammatory response up to 28 days post exposure. The authors reported an initial, acute inflammatory response one day post exposure, and lower but still significantly elevated inflammatory cytokine levels up to 28 days post exposure, indicating both acute and prolonged IONP-induced inflammatory responses. The authors also reported an increase in the expression of genes related to inflammation and tissue damage (including heat shock proteins and matrix metalloproteinases) throughout the study’s time course, as well as significant formation of micro granulomas—a potential precursor to fibrosis. These adverse effects were primarily attributed to excessive oxidative stress, as indicated by a significant reduction in glutathione (GSH) in the lavage fluid.

This same group also evaluated Fe_2_O_3_ particles (209.4 nm agglomerates) with a needle-like shape in ICR mice. Mice were administered the particles via a single intratracheal instillation (0.5, 1, or 2 mg/kg, or about 0.01 to 0.04 mg/mouse). The authors were able to show that the particles remained in the lung even 90 days post exposure, with many particles having been engulfed by alveolar macrophages. A failure to completely remove foreign particles from the lung has the potential to contribute to a chronic or dysregulated inflammatory response. The highest dose used in this study led to significant infiltration of inflammatory cells into the lung (neutrophils and lymphocytes), as well as a dose dependent increase in lactate dehydrogenase activity (LDH), increase in chemokine secretion, and increase in antigen presentation related protein expression. Overall, the authors concluded that IONP exposure induced a Th1 polarized immune response in the lungs [26].

Sadeghi et al. [27] dosed Wistar rats with 20 or 40 mg/kg Fe_2_O_3_ (20 nm) via repeated intratracheal instillations (either 7 or 14 times, once every other day). The authors found signs of pulmonary inflammation and injury, including pulmonary emphysema, and an increased presence of neutrophils, eosinophils, and lymphocytes. The authors also noted elevated hepatic enzymes in the blood serum, which is indicative of hepatic cell injury and liver damage. They attributed this to excessive oxidative stress, as indicated by an increase in free radicals and a reduction in GSH in the lung tissue.

Srinivas et al. [28] exposed Wistar rats via head and nose only inhalation (4 h continuous exposure) to Fe_3_O_4_ (15–20 nm) at a reported actual aerosol concentration of 640 mg/m^3^ based on mass median aerodynamic diameter and geometric standard deviation within respirable range—much higher than the OSHA permissible occupational exposure limit. Lung burden following exposure was not measured. The authors assessed lung injury and inflammation up to 14 days post-exposure, and showed that IONPs were able to induce acute cytotoxicity and inflammatory responses, as indicated by increased LDH, neutrophil infiltration, and pro-inflammatory cytokines in both lavage fluid and blood. The inflammatory response was sustained out to 14 days post exposure, although it decreased in severity in a time dependent manner. Once again, the authors attributed these adverse outcomes to excessive oxidative stress, as indicated by a significant reduction in GSH and antioxidant enzyme activities within this same time frame [28]. However, due to the excessive concentration used in this study, the observed response may have been due to overloading the lungs with particle, which would compromise normal pulmonary clearance mechanisms.

IONPs may also have the capacity to alter a typical immune response. Ban et al. [29] compared nano (35 nm) and sub-micron (147 nm)-sized Fe_2_O_3_ particles at 100, 250, or 500 μg/mouse. The animals were exposed via intratracheal instillation both before and after sensitization with ovalbumin (OVA), and the Th2 immune response was assessed. The authors found that the allergic response induced by OVA was inhibited with the middle or high doses of IONPs, but was actually enhanced at the lowest dose used for treatment, whereas the submicron-sized particles at the lowest dose had no effect at all. The authors were only able to conclude that the pulmonary immune response is sensitive to IONP exposure.

Gustafsson et al. [30] used a similar Fe_2_O_3_ particle (30 nm) in conjunction with the OVA sensitization model to further elucidate how IONPs may alter the immune response. In this case, the authors showed that intratracheal instillation of Fe_2_O_3_ alone (2.5 mg/kg body weight) induced a pulmonary inflammatory response in non-sensitized or OVA challenged Balb/c mice, as indicated by an increase in neutrophils, eosinophils, and lymphocytes in the airways. However, OVA-sensitized and challenged mice exposed to IONPs had a decreased inflammatory response. The authors attributed this to excessive cell death in already inflamed airways and in the lung draining lymph nodes (LDLN), most likely caused by excessive reactive oxygen species (ROS) generation in the resulting pro-oxidative environment.

Overall, these studies clearly indicate that IONPs have the capacity to induce lung inflammation and injury in both mice and rats, and that this is most commonly attributed to IONP-induced oxidative stress.

### 3.2. Pulmonary Fibrosis

Chronic inflammation has the potential to lead to pulmonary fibrosis, which is characterized by an increase in proliferation and collagen production by alveolar fibroblasts. This can be triggered by inflammatory cytokines such as TGF-β, and will normally occur following a foreign particle exposure or other type of lung injury to help initiate lung healing [40]. However, excessive or dysregulated fibrosis may lead to irreversible lung scarring, and have an impact on overall breathing capacity and oxygen intake. Based on the known capacity for IONPs to induce a pulmonary inflammatory response that has the potential to become chronic, it would seem logical that these particles may also have some sort of fibrotic capacity. Although very few studies exist, those summarized below show a clear potential fibrotic capacity of IONPs. Furthermore, these studies largely agree on oxidative stress and particle overload as being primary instigators of this potential adverse outcome.

Zhu et al. [31] used a single intratracheal instillation of Fe_2_O_3_ particles (22 or 280 nm) to expose Sprague Dawley rats to 0.8 and 20 mg/kg body weight and assessed subsequent adverse outcomes. One day post-exposure, the animals showed a peak in inflammation, as indicated by an increase in immune cell infiltration. By day 30, the animals exhibited markers of pro-fibrosis and lung emphysema, an increase in microvascular permeability, cell lysis, and other indicators of lung injury. The authors noted particle overloaded alveolar macrophages, and, again, attributed the observed adverse outcomes to an IONP-induced oxidative stress response.

Szalay et al. [32] used a single intratracheal instillation in Wistar rats (1 or 5 mg/kg body weight) of Fe_3_O_4_ (<50 nm diameter) and assessed potential fibrotic capacity of these particles. The authors reported a weak pulmonary fibrosis 30 days post exposure, but found no changes in extra-pulmonary organs.

A caveat to both of these studies, however, is that IONPs fibrotic capacity was only assessed up to 30 days post exposure, while most *in vivo* studies assess fibrotic outcomes after a longer time period. Therefore, while the above studies clearly demonstrate the pro-fibrotic capacity of IONPs, later time points are necessary to determine this more conclusively. Not only are there very few chronic *in vivo* IONPs studies, but the ones available do not include pulmonary fibrosis as a measured endpoint. This represents a clear and significant knowledge gap in the IONPs toxicity profile.

### 3.3. Genotoxicity and Carcinogenic Potential

The potential genotoxicity of IONPs is also inconclusive. Some studies report no genotoxic effect of IONPs [41], although study design issues make it difficult to conclusively determine this [42]. Furthermore, there are very few papers that assess this end point at all. This is likely due to a combination of factors, including the extended period of time it may take for tumors to develop in an *in vivo* model system, as well as the perceived benign quality of iron oxide dust [43]. However, pulmonary response after an extended period of time is a major concern for workers in an occupational setting who may be exposed to a particle over months or years, but may not experience cancer-related symptoms until much later. As mentioned previously, some studies involving fine or unknown-sized iron oxide particulates show an association between iron oxide exposure and lung cancer development in miners and other industrial workers. An overload of free iron in the lungs (siderosis) is also known to be associated with increased risk of cancer development [44]. Genotoxicity is often a crucial first initiating step in cancer development, and is an important endpoint that can be used to evaluate carcinogenic potential *in vivo*.

Totsuka et al. [33] used an intratracheal instillation of Fe_3_O_4_ (10 nm primary particle size) to expose both ICR and gpt delta mice at 0.05 or 0.2 mg per animal. Three hours post-exposure, the ICR mice showed signs of significant lung damage [33], and 24 h post exposure they had significantly increased formation of DNA adducts (DNA covalently bound to carcinogenic compound) with elevated etheno-deoxycytidine (ϵdC) levels. ϵdC is an indicator of inflammation and oxidative stress [34]. Eight weeks post-exposure, the gpt delta mice had increased gpt mutations (another indication of DNA damage), lipid peroxidation-related DNA adducts, inflammatory cell infiltration, and the formation of focal granulomas [33]. These results clearly indicate the potential for IONPs to induce genotoxicity, even within a very short time frame, and at a relatively low dose.

Genotoxicity is generally believed to be a significant driver of tumor development. However, there have been no *in vivo* studies that assess the actual carcinogenic potential of these particles. Even older *in vivo* cancer studies, which utilized an unknown or unreported-sized iron oxide, are scarce. Campbell et al. [45] exposed groups of 8–10 mice to 0.5 g Fe_2_O_3_·H_2_O for six hours a day, five days a week, over an entire year to mimic an occupational exposure scenario. At 800 days post exposure, the iron oxide-exposed mice had significantly increased formation of primary lung tumors as compared to control animals. However, the iron oxide particles were not characterized, and their actual size is unknown. Particularly with regards to IONPs, the potential carcinogenicity has not been well explored, and indicates a very clear knowledge gap in IONP-induced adverse effects.

### 3.4. Extra-Pulmonary Effects

IONPs, due to their small size, may be able to translocate to other organ systems following pulmonary exposure either as intact particles or as its solubilized constituents. Radioactively labeled IONPs have been shown to enter the circulating blood stream less than 10 min following a pulmonary exposure [35] and ultimately end up in organs which play a role in the mononuclear phagocyte system, including the liver, spleen, and kidney. This may lead to extra-pulmonary effects via the same or different mechanisms as IONPs pulmonary adverse effects. Once at these secondary sites, IONPs may dissolve, or may accumulate as whole particles and induce tissue damage. Furthermore, IONPs have been shown to activate or alter components of the blood such as platelets [46], and as was previously alluded to, may activate the immune system as well [29,30].

Al Faraj et al. [36] used magnetic resonance imaging to document superparamagnetic Fe_2_O_3_ translocation after an intrapulmonary administration. The particles (129.3 nm) were coated with polyethylene glycol and had either positively- or negatively- charged functionalization groups to enhance biocompatibility. After intrapulmonary administration into Balb/c mice at a calculated dose of 0.8 mmol iron/kg body weight, the authors looked for nanoparticles in several major organ systems at two hours, one day, two days, one week, two weeks, and one month post-exposure. They showed rapid movement of the particles to the liver at one day post-exposure, but this hepatic iron was almost completely cleared by later time points. The authors also showed that inflammatory biomarkers, lipid peroxidation, and DNA damage all increased with IONPs exposure as compared to non-treated control animals. Although previous studies showed that IONPs may persist in the lungs, this study indicates that if the particle translocates to a secondary site, it may be more rapidly cleared from there.

Wang et al. [37] used intratracheal instillation of 30 nm Fe_2_O_3_ into Wistar rats (twice per day for 3 consecutive days at 8.5 mg/kg body weight) and evaluated particle persistence in the lung, as well as translocation to, and persistence in, the liver. They showed significant increase in overall iron content in both the lung and the liver, as well as clear indications of IONP-induced lung and liver tissue damage, as determined via histopathology. The authors do not, however, assess the source of the elevated iron in the lung and liver. It could be from IONPs translocation, dissolution, and release of free iron ions, or may be due to IONPs sequestration of iron from the mitochondria or other organelles, leading to a cellular compensatory response.

Overall, the existing *in vivo* literature shows that pulmonary exposure to IONPs may lead to lung inflammation, injury, and immune modulation, with some studies also suggesting pro-fibrotic or fibrotic potential. There are conflicting studies as to the mutagenic potential of IONPs, with only one study showing actual tumor development (using an unreported size iron oxide). IONPs have also been shown to rapidly enter the circulatory system, potentially inhibiting platelet activation [46], as well as traveling to other major organ systems and causing tissue damage at these secondary sites.

Although *in vivo* studies provide information on potential IONP-induced adverse outcomes, there are also several significant knowledge gaps, including fibrotic potential and carcinogenic capacity. This lack of information makes it difficult to evaluate risk to a human population, and prevents a clear understanding of the IONPs toxicological profile.

## 4. Toxicity of IONPs—*In Vitro* Studies

Clearly, there is a critical need for a more complete toxicological profile of IONPs. In tandem, there is a dramatic increase in sheer number of new types of other nanomaterials, with similar knowledge gaps as to their unique potential to induce adverse outcomes. Overall, it is becoming increasingly urgent that more rapid methods are developed to better assess potential toxicity of these emerging materials, while concurrently maintaining robust and rigorous scientific methods to ensure these models are relevant and predictive of an *in vivo* and human response. In order to do this, many researchers are pushing for better *in vitro* model development, which would allow for a useful tiered hazard assessment of nanomaterials, in general.

There are many advantages to using *in vitro* model systems for toxicity assessment. In addition to the potential for more rapid toxicological evaluation, these models can also provide more information on the potential mechanism or mode of action of particle-induced toxicity. However, this can only be done if these *in vitro* model systems are predictive and representative of a human exposure and response. The overall goal with these systems, in the context of a tiered toxicity testing approach, is to utilize *in vitro* methods to rapidly screen a large number of nanomaterials for specific end points or adverse outcomes, also known as a high-throughput screening approach. These results can then be used to prioritize the most potentially hazardous materials for further *in vivo* studies.

However ideal this situation may seem, there are several issues with nanoparticle *in vitro* toxicity testing, which prevent this from being fully realized, and the study of IONPs *in vitro* toxicity is representative of these issues. Some studies report essentially no cytotoxic effect with exposure [47,48,49], whereas others report major dysfunction in macrophages [50] and mitochondria [51], and even DNA damage [52,53,54] induced by these particles. This lack of agreement is likely due to overall issues with *in vitro* model system experimental design and exposure conditions, and ultimately prevents them from being useful for hazard assessment purposes. In order to better utilize cell culture systems as even an initial screening tool, these major issues must be recognized and systematically addressed.

In the following section, this review will first discuss important considerations when developing an *in vitro* model system, including dose, particle agglomeration, particle uptake, cell type, and relevant end points, which may all have an impact on observed adverse outcomes. Next, this review will use these established criteria to critically examine the existing IONPs *in vitro* literature, and will highlight occupationally relevant IONPs *in vitro* studies which appear to correlate well to previously discussed *in vivo* results. All referenced *in vitro* studies are summarized in Table 4.

### 4.1. Issues with In Vitro Model Systems

#### 4.1.1. Particle Dose

An issue common to both *in vitro* and *in vivo* experimental design is the use of a relevant dose. With *in vivo* studies, based on mode of exposure and observed particle deposition, the delivered dose can be determined and compared to actual human burden to ensure dose relevancy. With *in vitro* studies, however, there are several other factors which will influence how much of the administered dose is actually delivered to the cells, and in what time frame that exposure may occur. Furthermore, a confounding factor specific to IONPs and other emerging nanomaterials is the lack of knowledge of what constitutes an occupationally relevant dose. There are, currently, only two studies which show actual human exposure to IONPs in an occupational setting (0.04–0.28 mg/m^3^ [3] or 0.083 mg/m^3^ [4]) and a much higher OSHA permissible exposure limit (14 mg/m^3^ over 8 h [5]).

Because the OSHA permissible exposure limit is likely to be the worst case exposure scenario, some researchers have focused on this dose specifically, and used it to better assess the translation from occupationally relevant animal exposure to *in vitro* model system. For example, Teeguarden et al. [55] exposed Balb/c mice to a 12.8 nm Fe_3_O_4_ particle via inhalation. Mice were exposed to 19.9 mg/m^3^ particle over 4 h, which is less (80 mg·h/m^3^) than the OSHA permissible exposure limit for fine-sized Fe_2_O_3_ particles (14 mg/m^3^ over 8 h or 112 mg·h/m^3^). After this inhalation exposure, the authors assessed target tissue dose in the bronchial region (about 1 μg/cm^2^), and the alveolar region (0.003–0.13 μg/cm^2^) of the lung. Based on these results, it would seem reasonable that an *in vitro* model system using cells derived from the bronchial region of the lung would require a higher dose of particle than cells derived from the alveolar region of the lung in order to obtain results that would be most representative for the deposited dose used in an *in vivo* exposure.

Although their findings have not necessarily been taken into account with other *in vitro* studies, this paper is an important guideline to use when evaluating *in vitro* studies and determining their potential relevance to an occupational exposure scenario.

#### 4.1.2. Particle Aggregation, Agglomeration, and Assay Interference

Even if particle deposition is taken into account, the administered *in vitro* dose may still not be representative of the delivered dose that reaches the cells. Agglomerate size, liquid media density, media protein content, and other factors will affect particle settling rate and dosimetry. This, in turn, may affect the administered to delivered dose ratios, and may have a major impact on subsequent adverse outcomes [56].

In fact, particle aggregation and agglomeration have already been shown to play a major role in the severity of IONP-induced adverse outcomes. As previously mentioned, it is generally accepted that smaller particles are able to induce more toxicity on a per mass basis due to increased surface area and particle number [22,23]. However, if these particles agglomerate in an unanticipated way, the observed adverse outcomes may be different than predicted. This would especially be the case if the agglomerate was recognized by the cell as a larger structure than the primary particle size suggests. This may not only lead to inaccurate toxicity assessment, but may also obscure potential size dependent particle toxicity.

For example, Karlsson et al. [48] showed no size-dependent toxicity when comparing “nano” and “sub-micron”-sized Fe_2_O_3_ and Fe_3_O_4_ particles—even with a relatively high dose (40 μg/cm^2^) administered to A549 cells. This is most likely due to particle agglomerate size. The primary particle size of the “nano” Fe_2_O_3_ was 30–60 nm, but once in suspension become about 1600 nm, making it very similar in size to the 150–1000 nm “sub-micron” Fe_2_O_3_. Similarly, the “nano” Fe_3_O_4_ had a primary particle size of 20–40 nm, but became greater than 200 nm as an agglomerate in suspension, again making it almost the same size as the 100–500 nm “sub-micron” Fe_3_O_4_ used. In fact, other researchers have shown that the agglomerate size of Fe_3_O_4_ (12.1 nm primary particle size) will vary significantly (199–453 nm) based on FBS content and overall density of the dosing media [57].

Additionally, the pH of the dosing media may have an impact on IONPs capacity to generate ROS [58]. The pH dependent dual enzymatic activity of IONPs may also obscure results if the dosing media used is not physiologically relevant. It is, therefore, critical that IONPs—and other nanomaterials, as well—are fully characterized in the media or vehicle that will be used for treatment.

Another factor that may interfere with perceived IONP-induced adverse outcomes is assay interference. This is relatively common due to the small size and pigmented properties of IONPs. For example, Kain et al. [53] noted that Fe_3_O_4_ particles (20–40 nm primary particle size, agglomerates >200 nm) caused DNA damage in Beas2B cells after 4 h of exposure to 20 μg/cm^2^ when this damage was measured via the comet assay, but that no oxidative DNA damage could be detected when formamidopyrimidine DNA glycosylase (FPG) sites were used as an indicator instead. The authors attributed this to IONP interference with FPG, leading to inaccurate results. Overall, the authors concluded that multiple methods should be used to verify results if particle interference is unknown with a particular assay. In fact, many different types of IONPs have been shown to interact with the components of cytotoxicity assays [59] and cytokine secretion assays [60], and likely interfere with others which have not yet been reported.

#### 4.1.3. Relevant Cell Type

In the lung, response to foreign particle exposure relies on chemical interactions and communications between a vast array of cell types. Due to the complexity of this system, it can be difficult to replicate this using cell culture models. There are several types of *in vitro* model systems that have been used, including mono-culture (one cell type), co-culture (two cell types), multicellular spheroid model systems, and even “organ-on-a-chip” methods. Although each of these model systems has clear advantages and disadvantages, one of the most necessary criteria that should be taken into consideration, is the use of a relevant cell type, including relevant disease state, species, and location in the body. This is also highly dependent on the overall goal of a study. For example, if the goal of a study is to assess IONPs response in a healthy individual, only cell lines derived from a healthy individual should be used. If the goal of a study is to assess IONPs impact on a particular endpoint, it is important to fully consider which cell types may be involved in this process, and which will allow for the most relevant assessment of a human response.

It is also important to consider the species of origin to determine if a cell line is going to be useful for a particular study. Although *in vivo* studies are typically used to establish potential risk following exposure to humans, and a good indicator of the success of an *in vitro* study is its correlation to *in vivo* results, it is also crucial to consider the differences between human and animal response with IONPs exposure. A mouse cell line is likely to be the best predictor of an *in vivo* mouse response, but a human cell line may be more predictive of a human response. Therefore, it is exceedingly important to consider mouse compared to human response both when designing cell culture-based systems, and for *in vivo* and *in vitro* experimental design.

It is also important to match organ location of a cell line used *in vitro* to the exposure scenario and end point being studied, so as to ensure the most predictive and relevant results. The same cell type from the same species but originating from two different organ systems may have an extreme impact on response following IONPs exposure. For example, mouse alveolar macrophages are known to have relatively low lysosomal activity as compared to mouse peritoneal macrophages. Park et al. [61] showed that this difference has a subsequent impact on IONP-induced adverse outcomes. The authors exposed murine alveolar macrophages at a range of doses from about 1.89 to 15 μg/cm^2^ of Fe_2_O_3_ (102 nm in cell culture media). After 24 h and at the highest dose used, they found a 40% decrease in cell viability, a 25% decrease in ATP production, and a 2 fold increase in both ROS and nitric oxide production as compared to non-treated controls. However, during a previous study which used the same nanoparticle but different cell line (mouse peritoneal macrophages) the authors saw a 20% decrease in cell viability, 40% decrease in ATP production, a 5 fold increase in ROS, and a 2.5 fold increase in nitric oxide as compared to non-treated controls [62]. They concluded that this difference between studies was most likely due to the differences in lysosomal activity and other characteristics which differentiate mouse alveolar macrophages from mouse peritoneal ones.

Clearly, the use of a relevant cell type is crucial for relevant and predictive results. However, very few IONPs studies utilize an *in vitro* model that is well matched to a specific end point and exposure scenario. This is likely a major contributor to the lack of cohesion or agreement on IONP-induced adverse outcomes based on *in vitro* studies.

### 4.2. Underlying Mechanisms of IONP-Induced Adverse Effects

By limiting IONPs literature to those studies which utilize relevant parameters based on the discussion above, *in vitro* IONPs studies appear to correlate well to observed *in vivo* outcomes. *In vitro*, IONPs have been shown to induce oxidative stress and a decrease in cell viability, as well as genotoxicity and neoplastic-like cellular transformation. These may all be components of, or precursors to, inflammation, pulmonary fibrosis, and potential carcinogenic capacity.

Oxidative stress and cell viability are basic assessments of potential particle toxicity, and also provide information as to the potential inflammatory capacity of a particle. Bhattacharya et al. [52] showed that a dose of 2 μg/cm^2^ (roughly comparable to Teeguarden et al. [55] established IONPs deposition in the bronchial region) of Fe_2_O_3_ (50 nm) induced a 15% decrease in cell viability, as well as elevated ROS generation in human bronchial fibroblasts (IMR-90). A study done with a less relevant, but still of human lung origin, cell line showed similar results with Fe_3_O_4_ (174 nm agglomerate). At about 3.03 μg/cm^2^, Dwivedi et al. [51] showed that IONPs induced a decrease in cell viability and indications of oxidative stress, including an increase in lipid peroxidation and a decrease in GSH in A549 cells.

As with *in vivo* work, one area in which *in vitro* nanotoxicology is generally lacking is in the assessment of carcinogenic potential of emerging nanomaterials. The major difficulty with this particular endpoint is that cancer is a multi-step, multi-cellular process that usually occurs over extended time periods. Therefore, acute *in vitro* exposures are unable to provide sufficient information about this particular area. However, there is some *in vitro* support for IONP-induced genotoxicity, and the potential development of a cancer-like phenotype.

IONPs have been shown to cause DNA damage using relevant experimental parameters as described above. Bhattacharya et al. [52] showed 10 and 50 μg/cm^2^ doses of Fe_2_O_3_ (50 nm) can cause double stranded DNA breaks (as measured via the comet assay) in both IMR-90 and Beas2B cell lines. Watson et al. [63] also showed the potential genotoxicity of IONPs using a novel CometChip assay, which functions similarly to the comet assay, but also takes into consideration potential particle interference as described above. Using this innovative platform, the authors showed that Fe_2_O_3_ (19.7 nm primary particle size, 934–1444 nm agglomerates) induced significant double stranded DNA breaks as compared to non-treated control cells, at doses of about 3.03 μg/cm^2^. IONP-induced genotoxicity, as shown in both of these studies, may lead to neoplastic-like cellular transformation, which has been separately shown to be induced by IONPs exposure.

Sighinolfi et al. [54] used Fe_3_O_4_ particles (161 nm) in the Balb/3T3 cell transformation assay at a dose of about 2.76 μg/cm^2^. This assay measures cellular transformation in mouse fibroblast cells and compares transformation rate induced by the particles to that of known tumor promoters. The authors found that IONPs exposure induced toxicity after 72 h, and was also able to promote tumoral foci, although they could not initiate them. These tumoral foci appeared to develop in conjunction with large IONPs agglomerates, and the authors noted that these agglomerates could potentially serve as a scaffold for foci engraftment [54]. It should be noted, however, that since most tumors are of epithelial origin, the use of fibroblasts in this example to assess the tumorigenic potential of a particle should be taken with caution.

Our research group developed an epithelial cell-based assay by using primary small airway epithelial cells to assess the capacity of Fe_2_O_3_ (12 nm primary particle size) to induce a neoplastic-like cellular transformation [64]. In this study, a low-dose sub-chronic exposure model (0.6 μg/cm^2^ continuously for 10 weeks) was used to mimic an occupationally relevant exposure scenario. After 10 weeks, the IONPs treated cells underwent a significant and dramatic neoplastic-like cellular transformation, as indicated by an increase in cell proliferation, formation of attachment-independent colonies, and immortalization. Overall, there is early evidence to suggest that IONPs can induce genotoxicity, and may cause a neoplastic-like cellular transformation under occupationally relevant exposure conditions.

**Figure 2:**
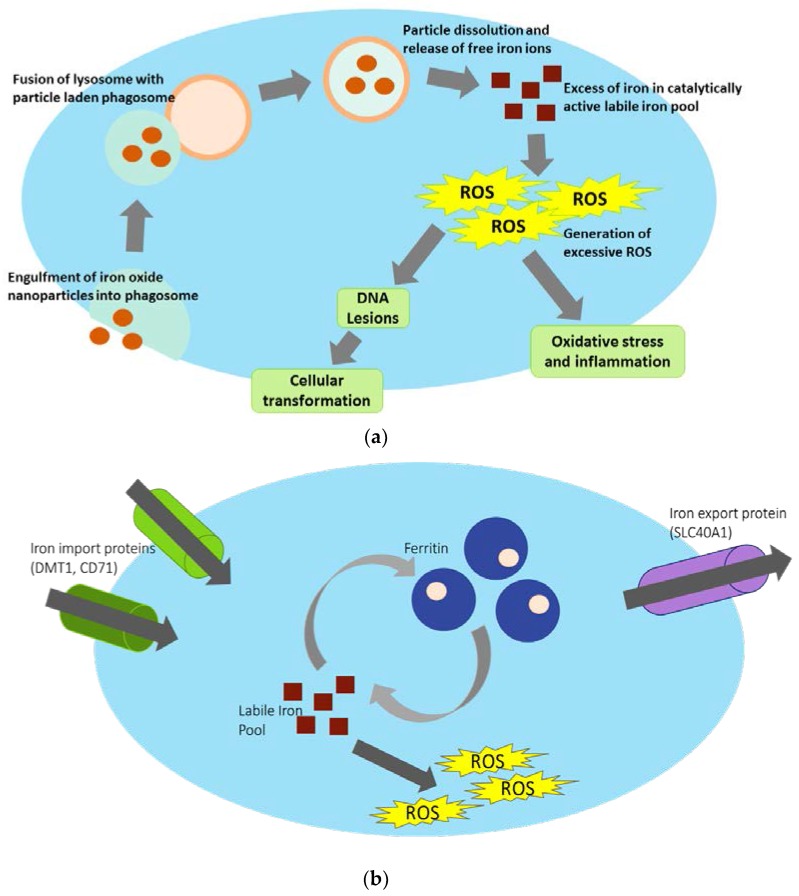
(**a**) Proposed mechanism behind IONP-induced iron homeostasis disruption and subsequent adverse outcomes. If IONPs are engulfed by the cell via phagocytosis and end up in a phagosome, this membrane bound vesicle will then fuse with the lysosome, creating an acidic environment for degradation. However, once in this acidic environment, IONPs will instead dissolve, releasing free iron ions into the cell’s catalytically active labile iron pool. This may ultimately result in increased and excessive ROS generation, and subsequent adverse outcomes. Dissolved particle may also affect iron stores in mitochondria and other organelles (not shown). (**b**) Maintenance of iron homeostasis via iron import proteins, iron storage proteins, and iron export proteins. In order to maintain appropriate iron levels within a cell, a complex network of iron-related proteins are involved in iron import, storage, and export. Example proteins for these processes (DMT1, CD71; ferritin; SLC40A1, respectively) are shown). Free Fe^2+^ in the labile iron pool is necessary for cellular function, but too much can cause an excess of reactive oxygen species generation via participation in the Fenton reaction. The labile iron pool is carefully maintained using iron storage mechanisms via ferritin. In pathologic conditions, increased iron in LIP will generate an excess of free hydroxyl radicals and induce adverse outcomes within the cell.

A critical question underlying the IONPs toxicity profile is the mechanism behind IONP-induced adverse effects. By again limiting literature to studies that utilize relevant parameters, as discussed above, IONP-induced oxidative stress appears to be a critical component of observed adverse outcomes both *in vitro* and *in vivo*. A proposed mechanism to this is heavily reliant on particle uptake, dissolution, and release of free iron ions into the cell’s catalytically active labile iron pool (Figure 2a). If IONPs are engulfed via phagocytosis, they will end up inside a phagosome. These phagosomes can then fuse with a lysosome, so the resulting acidic environment will degrade its contents. IONPs, however, may instead dissolve, releasing free iron ions (Fe^2+^) into the cell’s cytoplasm [65]. This catalytically available iron [66] can then participate in the Fenton reaction and generate ROS [67], an excess of which can ultimately induce DNA damage, lead to an inflammatory response, and/or carcinogenic related outcomes. In fact, Malvindi et al. [68] were able to show that Fe_3_O_4_ (32 nm) induced adverse effects on A549 cells (3 μg/cm^2^), which included decreased cell viability and increased LDH, ROS, and DNA damage. However, these outcomes were largely ablated with the use of an iron chelator. This clearly indicates that a primary source for IONP-induced damaging effects may be due to excessive free iron ions causing an oxidative stress response. 

Furthermore, Park et al. [61] were able to show that the difference in solubility between Fe_3_O_4_ and Fe_2_O_3_ nanoparticles could be linked to the severity of subsequent adverse outcomes—less soluble Fe_2_O_3_ also had a less severe impact on murine alveolar macrophages as compared to the more highly soluble Fe_3_O_4_. Additionally, Freyria et al. [49] clearly connected the mild solubility and poor reactivity of α-Fe_2_O_3_ to the almost negligible adverse outcomes observed following exposure, which included no cytotoxicity, necrosis, DNA damage, nor nitrate release in several cell types, regardless of particle size (87, 238, or 1100 nm) or high dose used (20–100 μg/cm^2^). Overall, these studies clearly show the demonstrable link between IONPs dissolution and subsequently observed adverse outcomes.

Other studies also suggest that IONPs may be involved in the sequestration of iron from mitochondria or other organelles, causing cellular compensation and prompting an influx of free iron ions into the cell’s labile iron pool [69]. Again, this would result in an overall excess of catalytically available iron, which can then participate in the Fenton reaction, and lead to increased ROS production within the cell as previously discussed. Overall, both proposed mechanisms remain largely unstudied, likely due to difficulty in differentiating the source of this catalytically available iron, as well as the complex network of proteins that are involved in maintaining the cellular iron homeostatic balance (Figure 2b).

Iron homeostasis is carefully regulated within a cell, particularly because iron is the major oxygen carrier in humans and is crucial for electron transfer and normal cellular functions. Catalytically active iron, which is diffusible and able to participate in redox reactions, is localized within a cytosolic labile iron pool [44]. Due to the sheer amount of available and inhalable iron in the atmosphere, the lung relies on the protein Divalent Metal Transporter 1 (DMT1), which is involved in the import of free iron into the cell. There, it can then be sequestered in the iron storage protein Ferritin (FTH) to limit its capacity to generate free radicals. Free iron may also be bound to the transferrin protein, which can then be imported into the cell via the transport protein CD71. Ultimately, iron may be exported out of the cell via Ferroportin (FPN) or a ZRT-IRE like protein (ZIP14) [70]. Expression levels of DMT1, FPN, and ZIP14 have all been shown to increase with an influx of iron content [71,72,73] to help combat potential toxicity associated with excessive free iron.

Generally, the lung and other organs are well equipped to deal with potential iron overload, which is suggested by the complex network of proteins involved in maintaining iron homeostasis. However, if IONPs are able to disrupt this careful balance—either through directly dissolving and releasing iron into the catalytically active labile iron pool, or by affecting iron stores within the mitochondria or other organelles—IONPs have the potential to overload this protective pathway, leading to an overall excess of iron in the catalytically active labile iron pool. This iron overload has been linked to increased pathogenesis of atherosclerosis [73], and increased iron levels in the lung are also associated with increased rates of lung pneumonia and lung infection [74]. Furthermore, the excessive oxidative stress may induce mitochondrial damage, which could then have deleterious effects on the cell, including increased induction of autophagy [75,76], as well as overall respiratory disease and pulmonary dysfunction [77,78].

Based on this proposed mechanism of action and published literature, the key to IONP-induced adverse outcomes appears to be largely reliant on particle dissolution and release of free iron ions. If this aspect can be affected by altering IONPs design, it may be possible to design a less hazardous IONPs which retains the properties necessary for its inclusion in biomedical or other applications. This would be a core component of a “safe by design” hazard reduction or prevention strategy.

## 5. “Safe by Design” IONPs Hazard Reduction Strategies

“Safe by design” hazard reduction strategy is the idea that if a nanoparticle can be designed in a way which both enhances its properties utilized for specific applications while simultaneously reducing its toxicity, this may have overall positive outcomes on worker-related safety concerns. It most often involves the alteration of one or more physicochemical properties of the particle to help alleviate its toxicity without compromising its intended purpose. Dissolution rate, surface coating, and functionalization can all be altered, and have all been shown to have an effect on overall IONP-induced adverse outcomes. IONPs dissolution, which appears to be a key component of subsequent adverse outcomes, can be alleviated primarily by altering particle surface coating and functionalization, as will be discussed below.

### 5.1. Alterations in IONPs Physicochemical Properties May Reduce Hazard

#### 5.1.1. Particle Surface Coating and Functionalization

Nanoparticle surface coating and functionalization are major components of nanoparticles which allow them to be used for specific applications. The surface coating can be altered to increase biocompatibility [79], to enhance paramagnetic or superparamagnetic properties for clinical and therapeutic applications [80,81], or to enhance catalytic properties [82,83]. An increase in coating thickness (and subsequent overall nanoparticle size) will increase relaxation times, allowing for better imaging results [84]. Surface modifications, such as lipids or polymers, have also been shown to stabilize nanoparticles in suspension, and could potentially control *in vivo* translocation [79,80]. In tandem, a change in surface coating or functionalization has also been shown to reduce particle dissolution rate, and may reduce or prevent IONP-induced adverse outcomes by acting as a “shield” to prevent or delay particle dissolution in the lysosome [85,86].

Particle surface coating and availability of surface binding sites have been shown to affect Fe_2_O_3_ particle (31 nm)-induced cytotoxicity in both Beas2B and A549 cells [87]. Furthermore, other researchers have shown that different IONPs coating types (dextran, amino-alcohol glucose derivative, or citrate) will affect Fe_2_O_3_ (7 nm primary particle size) dissolution rate in a coating dependent manner once in an acidic, lysosome-like environment [86]. The addition of a polyacrylic acid (PAA) coating onto superparamagnetic IONPs (7 nm) also reduced the leaching of iron in a neutral pH cell culture media [88]. In this case, PAA-coated IONPs were also less likely to aggregate, and did not induce any significant cytotoxicity when used to treat rat mesenchymal stem cells.

Shukla et al. [89] evaluated chitosan oligosaccharide coated Fe_3_O_4_ particles (6 nm). This coating is known to increase hydrophilicity and biocompatibility for use in a wide variety of biomedical applications. Even at very high doses (about 156–1250 μg/cm^2^), the authors found reduced cytotoxicity (as measured via MTT assay and cell staining), decreased deformation of mitochondrial membranes, and decreased ROS production in A549 cells with the coated nanoparticles as compared to uncoated. Furthermore, TEM-EDX elemental analysis revealed that the coated nanoparticles underwent less degradation in the cells and had a slower release of iron ions.

Tian et al. [85] examined silica-coated and uncoated Fe_3_O_4_ particles (8 nm) in a lysosomal model system, and found that fewer Fe^3+^ ions were released with the coated nanoparticles as compared to uncoated ones. Furthermore, these coated particles were taken up by human mesenchymal stem cells at a similar rate to uncoated particles but were metabolized and excreted more slowly. This could potentially be of positive benefit for the particles to be better utilized in a biomedical setting.

Polymer functionalization may also have a direct impact on IONP-induced toxicity. For example, Cochran et al. [90] utilized a poly (trolox ester) coated iron oxide nanoparticle. The poly (trolox ester) was specifically designed to release trolox locally to help combat IONP-induced pro-oxidant effects. Their results showed that human endothelial cells (HUVECs) treated with about 9.375 μg/cm^2^ IONPs had significantly decreased cell viability and increased ROS generation than non-treated control cells. However, cells treated with a poly (trolox ester) coated iteration of the same nanoparticle showed similar viability and ROS generation as the non-treated control cells. The authors conclude that the addition of a polymer coating may be able to directly counteract IONP-induced oxidative stress related adverse outcomes. A major caveat with particle coating and functionalization toxicity assessment, however, is that there are an infinite number of coatings and functionalization combinations which could be used with IONPs, not all of which may be relevant for human health. Furthermore, there are many coatings that may impart novel function while reducing toxicity, but haven’t been developed or investigated yet.

Overall, these studies highlight that particle dissolution as related to surface coating and functionalization may play a major role in determining *in vitro* adverse outcomes following IONPs exposure. An alteration in these physicochemical properties could be used to help alleviate this toxicity, as well as to potentially enhance other properties that allow for IONPs use in a broad range of applications.

## 6. Summary

Currently, prevailing information on the toxicity of IONPs suggests potential risk associated with exposure, including inflammation, fibrosis, genotoxicity, and extra-pulmonary effects, all of which have been attributed to increased oxidative stress following exposure. The majority of this literature correlates particle internalization, dissolution, and subsequent release of free iron ions to be the primary source for these adverse outcomes. Some emerging studies show promise in the alteration of physicochemical properties of these nanoparticles (particularly surface coating and functionalization) to help prevent or reduce this toxicity. However, there is still little concurrence on its actual effectiveness, or the resulting impact on nanoparticle function. Therefore, more research involving long term *in vivo* exposure to assess particle distribution, and *in vitro* work that utilizes relevant parameters (such as dose, cell type, and relevant end points), must be conducted to better ascertain the risk associated with IONPs exposure. Furthermore, there is a major knowledge gap as to which physicochemical alterations could affect bioactivity and toxicity following exposure, and which ones could be altered to reduce this toxicity. Without this information, it is exceedingly difficult to make accurate recommendations for safe exposure limits. Despite this, IONPs continue to be incorporated into consumer products and can be released during the manufacturing process, or throughout its lifecycle, becoming a source of potential hazard for at-risk workers. Therefore, there is a critical need to better characterize the IONPs toxicity profile, and to establish more robust *in vitro* and *in vivo* toxicity assessment, so as to better assess risk for a wide range of nanomaterials and to protect at-risk workers.

## Figures and Tables

**Table 1:** Referenced iron oxide (unknown or unreported size) human studies.

Reference	Cohort	Exposure Particulates	Size	Adverse Outcomes
[6] Boyd	6000 underground and surface hematite iron ore miners (UK)	Iron oxide dust with 10–12% silica content, radon	Unknown	70% increased lung cancer mortality rate
[20] Siew	Employed males 1906–1945 (Finland)	Iron fumes/dust, welding fumes	Unknown	Increased risk of lung cancer following exposure to one or both types of particulates
[21] Chen	Underground iron ore miners and surface workers (Longyan and Taochong, China)	3.8 mg/m^3^ total airborne dust, 28% iron content	Unknown	Increased incidences of non-malignant respiratory disease and lung cancer
[10] Faulds & Stewart	Hematite miners decreased 1932–1953 (West Cumberland, UK)	Ferric oxide with 10–12% silica content	Unknown	Almost 5 fold increased incidences of lung carcinomas at time of death (attributed to silica content)

**Table 2:** Referenced iron oxide nanoparticle human studies.

Reference	Cohort	Exposure Particulates	Size	Adverse Outcomes
[24] Andujar	21 welders, average 27 years exposure	Iron oxide, manganese oxide, chromium oxide	Unknown/not-reported	Fibrotic lesions, elevated iron load. In vitro treatment with representative nanoparticles caused increased secretion of pro-inflammatory cytokines.
[4] Pelclova	14 workers in iron oxide pigment production facility, average 10 years exposure	Iron oxide (primarily α-Fe_2_O_3_)	80% measured particles less than 100 nm in diameter	Elevated oxidative stress and inflammatory biomarkers in exhaled breath condensate and urine.

**Table 3:** Referenced iron oxide nanoparticle *in vivo* studies.

Study	Animal	Particle	Primary Particle Size	Dose	Mode/Duration of Exposure	Time Points	Adverse Outcomes
[25] Park	ICR mice	Fe_3_O_4_	5.3 nm	0.25, 0.5, 1 mg/kg body weight	Intratracheal instillation	1, 7, 14, 28 days	Inflammation
[26] Park	ICR mice	Fe_2_O_3_	10 nm (209.4 nm agglomerate)	0.5, 1, 2 mg/kg body weight	Intratracheal instillation	90 days	Inflammation, Th1 polarized immune response
[27] Sadeghi	Wistar rats	Fe_2_O_3_	20 nm	20 or 40 mg/kg body weight	Intratracheal instillation (7 or 14 times, once every other day)	1 day post exposure set completion	Inflammation, liver damage
[28] Srinivas	Wistar rats	Fe_3_O_4_	15–20 nm	640 mg/m^3^	Inhalation, 4 h continuous	1, 2, 14 days	Inflammation
[31] Zhu	Sprague Dawley rats	Fe_2_O_3_	22 or 280 nm	0.8 or 20 mg/kg body weight	Intratracheal instillation	1, 30 days	Inflammation, pro-fibrosis, longer prothrombin and activated partial thromboplastin times
[32] Szalay	Wistar rats	Fe_3_O_4_	<50 nm	1 or 5 mg/kg body weight	Intratracheal instillation	1, 3, 7, 14, 30 days	Weak fibrosis
[33] Totsuka	ICR or gpt delta mice	Fe_3_O_4_	10–100 nm	0.05 or 0.2 mg/animal	Intratracheal instillation	3 h, 8 weeks	DNA damage in lungs, DNA adduct formation, inflammation, focal granuloma formation
[34] Ishino	ICR mice	Fe_3_O_4_	10–100 nm	0.2 mg/animal	Intratracheal instillation	1 day	DNA adducts (elevated ϵdC)
[45] Campbell	Mice (strain unknown)	Fe_2_O_3_·H_2_O	Unknown	0.5 g for 8–12 animals	Inhalation, 6 h/day continuous, 5 days/week, 1 year	Up to 800 days (or death of animal)	Primary lung tumors
[35] Zhu	Sprague Dawley rats	^59^Fe_2_O_3_	22 nm	4 mg/animal	Intratracheal instillation	Daily, up to 50 days	IONPs can pass into systemic circulation, and is distributed to mononuclear phagocyte rich organs
[36] Al Faraj	Balb/c mice	Fe_2_O_3_	129.3 nm	0.8 mmol iron/kg body weight	Intrapulmonary administration (once or three times on consecutive days)	2 h, 1 or 2 days, 1 or 2 weeks, 1 month	Particle translocation to liver, lipid peroxidation, DNA damage, inflammation biomarkers
[37] Wang	Wistar rats	Fe_2_O_3_	30 nm	8.5 mg/kg body weight	Dry powder nasal spray, twice daily for three days	Up to 36 h	Severe lung and liver tissue damage
[29] Ban	Balb/c mice	Fe_2_O_3_	35 or 147 nm	100, 250, or 500 μg/mouse	Intratracheal administration (four times) with or without OVA sensitization	24, 48 h after completion of exposure set	Inhibition of OVA-induced allergic response at high dose, enhancement with low dose
[30] Gustafsson	Balb/c mice	Fe_2_O_3_	30 nm	2.5 mg/kg body weight	Intratracheal instillation with or without OVA sensitization	1, 2, 7 days post exposure	Decreased inflammation with IONP and OVA attributed to excessive cell death in inflamed airways and lung draining lymph nodes
[55] Teeguarden	Balb/c mice	Super-paramagnetic IONPs	12.8 nm	19.9 mg/m^3^	Inhalation, four hour continuous	Up to 7 days	Particle deposition, interstitial inflammation, macrophage infiltration

**Table 4:** Referenced iron oxide nanoparticle *in vitro* studies.

Study	Cell Type	Particle Type	Primary Particle Size	Agglomerate Size in Dosing Media	Particle Dose (μg/cm^2^)	Adverse Outcomes
[57] Watanabe	A549	Magnetic Fe_3_O_4_	10 nm	197 nm	0.303–30.3 μg/cm^2^	Cell membrane damage, increased ROS and oxidative DNA damage, decreased GSH, increased CD44^+^ fraction and HO-1 expression
[48] Karlsson	A549	Fe_2_O_3_, Fe_3_O_4_	Fe_2_O_3_: 29 nm, <1 μm Fe_3_O_4_: 20–30 nm, 0.5 μm	Fe_2_O_3_: 1600 nm, 150–1000 nm Fe_3_O_4_: >200 nm, 100–500 nm Fe_2_O_3_: 102 nm Fe_3_O_4_: 26 nm	40 μg/cm^2^	Cytotoxicity, mitochondrial damage, DNA damage
[62] Park	Murine peritoneal macrophages	Fe_2_O_3_	NR	102 nm	1.95–15 μg/cm^2^	Cytotoxicity, decreased ATP production, increased ROS, nitric oxide, TNF-α secretion
[53] Kain	Beas2B	Fe_3_O_4_	20–40 nm	<200 nm	20 μg/cm^2^	DNA damage
[52] Bhattacharya	IMR-90, Beas2B	Fe_2_O_3_	NR	50 nm	2–50 μg/cm^2^	Cytotoxicity, DNA damage, increased ROS
[51] Dwivedi	A549	Fe_3_O_4_	36 nm	174 nm	3.03–15 μg/cm^2^	Cytotoxicity, increased ROS, decreased GSH and mitochondrial membrane potential
[54] Sighinolfi	Mouse fibroblasts	Fe_3_O_4_	20–50 nm	161 nm	2.76 μg/cm^2^	Promote tumoral foci, scaffold for foci engraftment
[64] Stueckle	pSAEC	Fe_2_O_3_	19 nm	341.56 nm	0.6 μg/cm^2^	Increased formation of attachment independent colonies
[68] Malvindi	A549	Fe_3_O_4_	32 nm	107 nm	3 μg/cm^2^	Cytotoxicity, increased LDH, ROS, DNA damage
[49] Freyria	A549, murine alveolar macrophages	Fe_2_O_3_	87, 238, 1100 nm	69, 357, 888 nm	1–100 μg/cm^2^	No effect on LDH, DNA damage, apoptosis/necrosis, extracellular nitrite
[89] Shukla	A549	Fe_3_O_4_, chitosan oligosaccharide coating	6 nm	NR	156–1250 μg/cm^2^	With coating: reduced cytotoxicity, decreased deformation of mitochondrial membranes, ROS production, decreased particle degradation and more controlled release of iron ions
[85] Tian	Human mesenchymal stem cells	Fe_3_O_4_, silica coating	8 nm	NR	100 μg Fe/mL (unable to determine equivalent μg/cm^2^ dose)	With coating: slower metabolism/particle excretion, less iron ion release in acidic environment
[88] Guldris	Rat mesenchymal stem cells	Super-paramagnetic IONPs, PAA coating	7 nm	18, 35 nm	Up to 62.5 μg/cm^2^	No effect on cell viability, cell injury/damage
